# Loss of Tau protein affects the structure, transcription and repair of neuronal pericentromeric heterochromatin

**DOI:** 10.1038/srep33047

**Published:** 2016-09-08

**Authors:** Zeyni Mansuroglu, Houda Benhelli-Mokrani, Vasco Marcato, Audrey Sultan, Marie Violet, Alban Chauderlier, Lucie Delattre, Anne Loyens, Smail Talahari, Séverine Bégard, Fabrice Nesslany, Morvane Colin, Sylvie Souès, Bruno Lefebvre, Luc Buée, Marie-Christine Galas, Eliette Bonnefoy

**Affiliations:** 1 Université Paris Descartes, Centre Interdisciplinaire Chimie Biologie-Paris, Inserm UMRS1007, Paris, France; 2 Université de Lille, Inserm, CHU-Lille, UMRS1172, Alzheimer & Tauopathies, Lille, France; 3 Laboratoire de Toxicologie Génétique, Institut Pasteur de Lille, Lille, France

## Abstract

Pericentromeric heterochromatin (PCH) gives rise to highly dense chromatin sub-structures rich in the epigenetic mark corresponding to the trimethylated form of lysine 9 of histone H3 (H3K9me3) and in heterochromatin protein 1α (HP1α), which regulate genome expression and stability. We demonstrate that Tau, a protein involved in a number of neurodegenerative diseases including Alzheimer’s disease (AD), binds to and localizes within or next to neuronal PCH in primary neuronal cultures from wild-type mice. Concomitantly, we show that the clustered distribution of H3K9me3 and HP1α, two hallmarks of PCH, is disrupted in neurons from Tau-deficient mice (KOTau). Such altered distribution of H3K9me3 that could be rescued by overexpressing nuclear Tau protein was also observed in neurons from AD brains. Moreover, the expression of PCH non-coding RNAs, involved in PCH organization, was disrupted in KOTau neurons that displayed an abnormal accumulation of stress-induced PCH DNA breaks. Altogether, our results demonstrate a new physiological function of Tau in directly regulating neuronal PCH integrity that appears disrupted in AD neurons.

Tauopathies are neurodegenerative diseases among which Alzheimer’s disease (AD) is the most frequent. The hallmark of all tauopathies is the histopathological appearance of intracellular neuronal aggregates of the microtubule-associated protein (MAP) Tau^1^. Under physiological conditions, Tau protein displays a high degree of intrinsically disordered conformation^2^ whose alteration induced by pathological hyperphosphorylation leads to its intracellular oligomerization and aggregation. There is a large consensus that Tau dysfunction, driven by hyperphosphorylation, is one of the main factors responsible for the neurodegenerative disorders associated with tauopathies^3^. However, whether the impact of Tau hyperphosphorylation on Tau-dependent neurodegenerative disorders is predominantly due to a toxic gain-of-function of hyperphosphorylated aggregated Tau and/or to a loss-of-function(s) of the physiological intrinsically disordered protein remains open.

Even though Tau has been defined as a MAP, other non-conventional subcellular distributions of Tau protein have been described. Interestingly, nuclear localization of Tau has been observed in neuronal and non-neuronal cells by different groups^4^. In previous work, we demonstrated in a mouse model that neuronal nuclear Tau protein plays an important role in preserving DNA and RNA integrity under ROS-inducing hyperthermia stress conditions^5^,^6^. In addition to its nuclear localization, Tau has been shown to interact with DNA through the minor groove and form protein DNA complexes^5^,^7^,^8^,^9^,^10^. In non-neuronal cells, Tau protein co-localizes with H3K9me2-rich DNA sequences positioned at the periphery of the nucleolus and forms protein-DNA complexes with the AT-rich major satellite sequences constituting murine pericentromeric heterochromatin (PCH)^7^ and PCH structures are altered in *Drosophila* and murine models of tauopathies^11^. Altogether, these results suggest a potential role for Tau protein in regulating PCH structures.

PCH is composed of highly repeated major satellite DNA sequences and displays a highly ordered nucleosome distribution rich in particular epigenetic marks such as the trimethylated form of lysine 9 of histone H3 (H3K9me3) and in specific proteins such as the heterochromatin protein 1α (HP1α) giving rise to compact chromatin regions that influence genome stability and gene expression regulation^12^,^13^,^14^,^15^,^16^. With the goal of further analysing the physiological role of Tau protein in maintaining neuronal genome structure and organization, we have addressed here the question of the physiological role of Tau protein with respect to the organization of PCH DNA regions. To this end, we have used primary cultured neurons from either wild-type (WT) or Tau-deficient (KOTau) mice. In addition to analysing the interaction of Tau with PCH major satellite DNA sequences, several parameters of PCH structure and function were investigated. Using electron microscopy and chromatin immunoprecipitation we show that a fraction of nuclear Tau binds to and localizes within or next to neuronal PCH DNA. Using immunofluorescence and quantitative single-cell imaging, we showed that the clustered distribution of H3K9me3 and HP1α co-localizing with chromocenters was disrupted in KOTau neurons compared with WT, although the global amount of H3K9me3 and HP1α remained unchanged. Such deregulation of PCH organization, which could be rescued by overexpressing nuclear hTau protein in KOTau neurons, was also observed in AD neurons that displayed pathological hyperphosphorylated Tau. Moreover, we observed that such disruption of PCH organization impaired the expression of non-protein coding RNAs transcribed from PCH and was associated with a high degree of DNA breaks accumulated at PCH sequences of KOTau neurons submitted to stress conditions.

## Results

### Nuclear Tau interacts with PCH in primary cultured neurons

**Figure 1:**
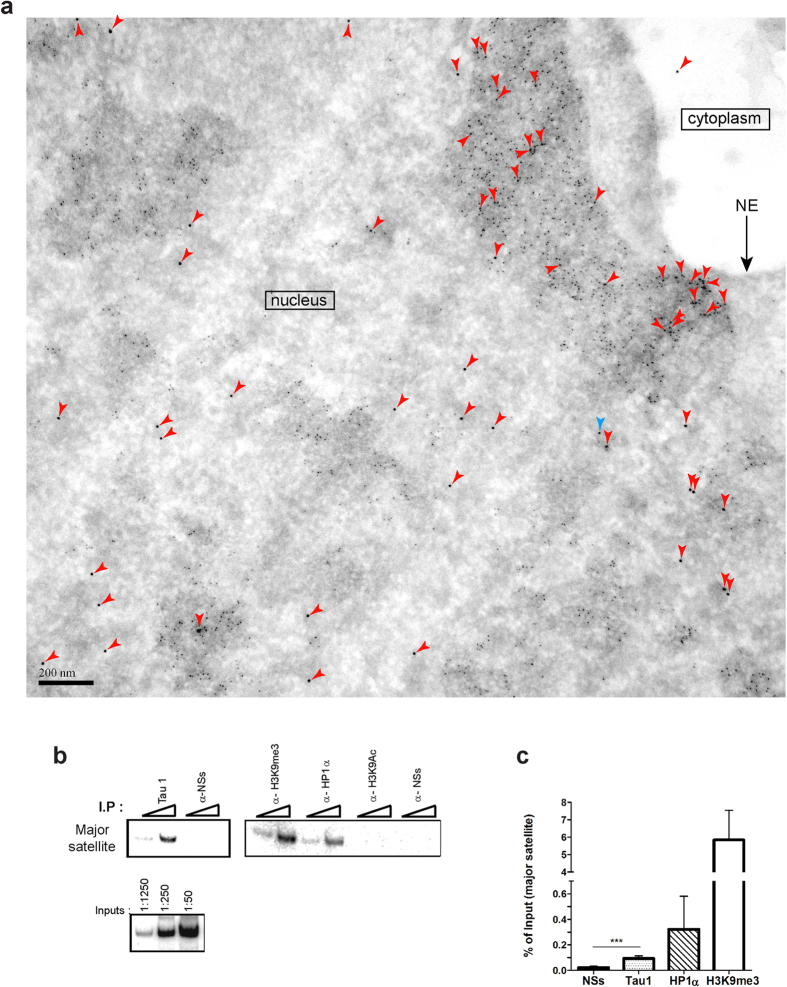
Tau protein interacts with H3K9me3-rich pericentromeric DNA regions. (**a**) Immunogold electron micrographs of nuclei from primary neuronal cultures of mouse embryo cortex indicate the presence of Tau protein labelled with Tau5 antibodies (12 nm gold particles indicated by red arrowheads) within or in the proximity of neuronal PCH densely labelled with anti-H3K9me3 antibodies (6 nm gold particles). Tau-immunogold particles were identified applying the Analysis Particles command of Image J software using the particle indicated by a blue arrowhead as the lower size limit for the 12 nm gold particles recognizing the Tau5 antibody. (**b**,**c)** Tau protein interacts with neuronal PCH murine major satellite sequences as revealed by ChIP assays of genomic DNA from primary cultured neurons immunoprecipitated with Tau1, anti-HP1α, anti-H3K9me3 and anti-H3K9Ac and anti-NSs (negative controls) antibodies with immunoprecipitated (I.P) and input DNA amplified by either (**b**) PCR or **(c)** qPCR using primers specific to murine major satellite DNA sequences. Quantification (**c**) was carried out from either n = 4 (in the case of Tau1 and anti-NSs antibodies) or n = 2 (in the case of anti-HP1α and anti-H3K9me3) independent chromatin immunoprecipitation assays. Data are means ± s.d. Student’s t-test, p-value = 0.0007 less than 0.001 (***). Bars = 200 nm.

To investigate the subnuclear distribution of Tau protein with respect to PCH in neurons, we carried out an immunoelectron microscopy analysis of neuronal nuclei from embryonic primary neuronal cultures using gold-labelled antibodies directed against H3K9me3 (small 6 nm gold particles), which is an epigenetic mark specifically concentrated within PCH DNA regions, or Tau5 antibody (large 12 nm gold particles indicated by red arrowheads) recognizing total Tau protein. As expected, H3K9me3 displayed a characteristic PCH distribution, concentrated within large electron dense heterochromatin DNA domains (Fig. 1a). In contrast to a previous immunoelectron microscopy analysis that showed Tau protein in neuronal nuclei solely under heat-stress conditions^5^, the results obtained in this study using Tau5 alongside anti-H3K9me3 antibodies clearly show that Tau protein is present in neuronal nuclei under physiological non-stress conditions. Notwithstanding the variability among heterochromatin clusters, Tau was often positioned either within or in close proximity to darkly stained heterochromatin domains labelled with H3K9me3 (Fig. 1a). To further confirm the capacity of Tau protein to interact with PCH in neurons under physiological conditions, we carried out chromatin immunoprecipitation (ChIP) assays of total neuronal DNA with antibodies directed against Tau unphosphorylated at epitope 195–202 (Tau1), previously detected in the nucleus^5^, as well as with antibodies against HP1α and H3K9me3 (positive controls) and against H3KAc9 (an epigenetic mark absent from PCH DNA sequences^15^) and viral NSs protein encoded by Rift Valley Fever Virus, absent in these cells used as negative controls. Immunoprecipitated DNA (I.P.) and total non-immunoprecipitated (input) DNA were amplified with primers specific for murine major pericentromeric satellite DNA sequences that constitute murine PCH^13^,^17^,^18^. As shown in Fig. 1b, Tau protein was found to be associated with the major satellite sequences as H3K9me3 and HP1α, whereas neither H3K9Ac nor NSs interacted with major satellite sequences indicative of Tau binding to H3K9me3- and HP1α-rich sequences constituting PCH.

The proportion of major satellite sequences interacting with Tau1, HP1α, H3K9me3 and NSs was then quantified by qPCR with respect to input. The results, shown in Fig. 1c, are indicative of a significant interaction of the unphosphorylated form of Tau protein with major PCH satellite DNA sequences as compared to NSs. In agreement with electron microscopy images that showed a higher proportion of H3K9me3 with respect to Tau5 labelling within electron dense PCH regions, the fraction of major satellite DNA sequences interacting with Tau was largely inferior to the fraction of major satellite DNA interacting with H3K9me3.

### Endogenous Tau protein regulates the clustered distribution of H3K9me3 in neuronal nuclei

**Figure 2:**
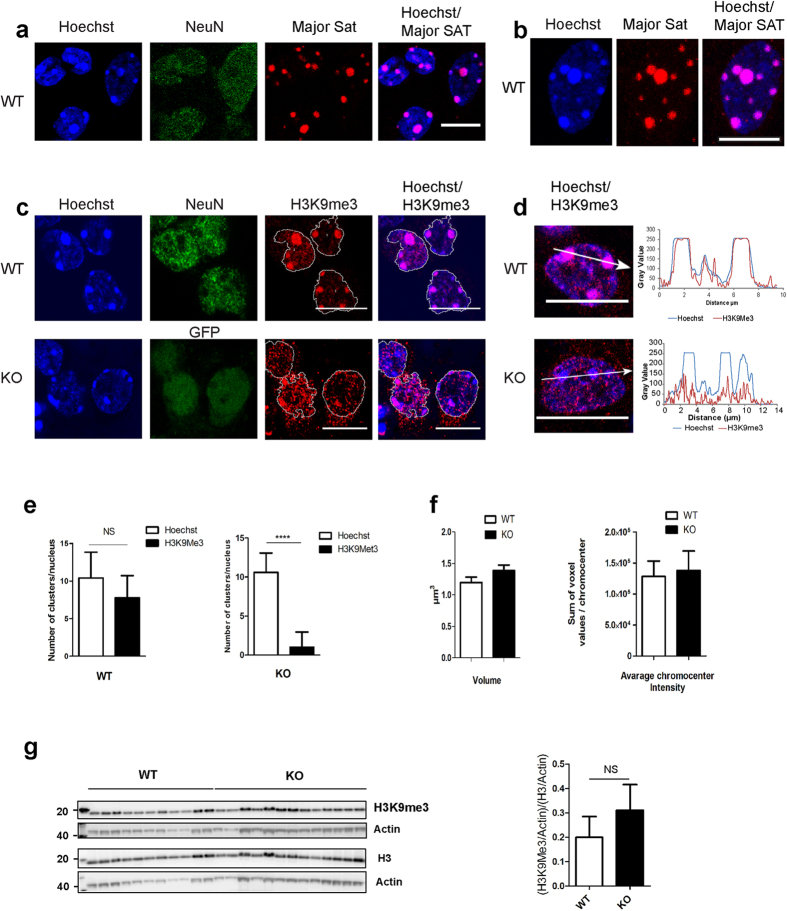
The clustered distribution of H3K9me3 within chromocenters is disrupted in Tau-deficient primary cultured neurons. (**a**) Single confocal section and (**b**) z-projection of all confocal sections of nuclei from primary culture of WT neurons labelled with Hoechst and anti-NeuN antibodies combined with FISH using a rhodamine-labelled murine pericentromeric major satellite probe indicate that in murine neurons, PCH major satellite DNA sequences are concentrated within nuclear sub-structures densely labelled with Hoechst corresponding to chromocenters. (**c**–**f**) The clustered distribution of H3K9me3 that co-localizes with chromocenters in WT neurons is disrupted in nuclei of KOTau neurons, as observed in (**c**) single confocal sections of nuclei from primary cultured WT or KOTau (KO) neurons (with nuclei outlined relative to Hoechst fluorescence) and as indicated by (**d**) the quantification of the fluorescence signals for Hoechst and H3K9me3 along the indicated line scans drawn across chromocenters in a confocal section of nuclei from WT or KOTau (KO) neurons. (**e**,**f**) The number of H3K9me3 clusters *per* nucleus but not the number, volume or intensity of chromocenters (corresponding to Hoechst clusters) is strongly decreased in KOTau neurons compared with that in WT neurons as quantified after 3D reconstruction of nuclei from three independent WT and KOTau primary cultures of neurons with n = 250 WT and n = 212 KOTau clusters analysed. (**g**) Western blot analysis of lysates from n = 11 WT and n = 14 KOTau independent primary cultures using antibodies against H3K9me3, H3 and actin indicate that Tau deficiency does not affect the level of neuronal H3K9me3. The level of H3K9me3 and H3 in each sample was estimated densitometrically with respect to the level of actin protein in the same sample. Data are means ± s.d. (**e**,**g**) ± s.e.m. (**f**). (**e)** Chi-square test with p-value < 0.0001 (****) and NS (non-significant). (**g**) Student’s t-test. Bars = 10 μm.

In the majority of murine cells, major satellite DNA sequences from different chromosomes come together to form specific nuclear sub-structures named chromocenters that are easily detectable under the microscope as regions densely labelled by DNA intercalating agents such as Hoechst. In primary cultured neurons, identified as neurons by their capacity to be labelled by anti-NeuN antobodies, chromocenters were also clearly visible as regions where major satellite sequences were concentrated, as indicated by fluorescence *in situ* hybridization (FISH) assay using a probe specific to major satellite sequences (Fig. 2a). Co-localization of all chromocenters with the major-satellite probe was clearly observed at the level of a single confocal section (Fig. 2a) as well as along the z-projection of all confocal sections (Fig. 2b).

The presence of H3K9me3 within chromocenters is a prominent and conserved hallmark of PCH that controls most of PCH functions^16^. To examine the potential role of Tau protein in PCH organization, we analysed the presence and distribution of H3K9me3 with respect to chromocenters in nuclei of primary cultures of neurons from either WT or KOTau (KO) mice by immunofluorescence and confocal microscopy.

Figure 2c shows a representative confocal section of either WT or KOTau nuclei labelled with the DNA intercalating Hoechst dye, an anti-H3K9me3 antibody and the corresponding merged images. In the case of primary cultures of neurons from WT mice, neurons were identified using anti-NeuN antibodies whereas in primary cultures from KOTau mice, neurons were identified by their capacity to express the gene coding for GFP inserted in the Tau genomic locus replacing the *Mapt* gene coding for Tau protein^19^.

In WT neurons, a clustered distribution of H3K9me3 was clearly visible to co-localize with chromocenters densely labelled with Hoechst whereas in KOTau neurons, H3K9me3 appeared abnormally dispersed throughout the nucleus and only weakly present or totally absent from chromocenters (Fig. 2c). Line scans were drawn across chromocenters to quantify the relative intensities of the fluorescence signals for Hoechst (blue) and H3K9me3 (red) (Fig. 2d). In WT neurons, the two intensity profiles overall overlapped, with H3K9me3 at a maximum across all the chromocenters whereas in KOTau neurons, H3K9me3 fluorescence was scattered alongside the line scan with only casual peak intensity at chromocenters. This analysis confirmed that in KOTau neurons H3K9me3 is mainly absent from chromocenters. Surprisingly, in KOTau neurons, H3K9me3 scattered in small clusters were detected not only in the nucleus but also in the cytoplasm (Fig. 2c). To determine the number of chromocenters (corresponding to Hoechst clusters) and H3K9me3 clusters per nucleus, a quantitative 3D analysis of Hoechst and H3K9me3 labelling was performed. In contrast to the number of chromocenters/nuclei in WT and KOTau neurons, which remained unchanged (≈10), the number of H3K9me3 clusters/nuclei dramatically diminished in KOTau (≈1) neurons with respect to the number observed in WT (≈10) neurons (Fig. 2e); the result was an average number of H3K9me3 clusters/nuclei that was significantly different from the average number of chromocenters/nuclei in KOTau but not WT neurons (Fig. 2e). As shown in Fig. 2f, the dispersed distribution of H3K9me3 observed in KOTau neurons affected neither the average volume nor the average intensity of chromocenters.

To determine whether the absence of a clustered distribution of H3K9me3 observed in KOTau neurons could be the consequence of a decrease in the total amount of H3K9me3 present in these cells, we next carried out western blot analysis of the total amount of H3K9me3 and H3 in WT and KOTau neurons from 11 and 14 independent cultures respectively. As shown in Fig. 2g, the ratio H3K9me3 to H3 not only did not decrease but displayed an increase in KOTau neurons with respect to WT neurons. Thus, Tau deficiency altered the organization of PCH leading to an abnormally dispersed distribution of H3K9me3 without interfering with the rate of histone H3 methylation into H3K9me3.

To confirm the role of the nuclear localization of Tau protein in regulating the clustered distribution of H3K9me3, we next analysed the capacity of cytoplasmic and nuclear Tau protein to rescue the H3K9me3-dispersed phenotype observed in KOTau neurons. To this end, KOTau neurons were infected with lentiviral vectors encoding for hTau0N4R protein (KOhTau) or Tau protein fused to a nuclear localization signal, hTau0N4R-NLS (KOhTauNLS). In neurons, several isoforms of Tau protein exist and previous profiling of Tau isoforms has shown that the major 0N isoform of Tau is almost exclusively found in the cell body and axons, in contrast to the 1N isoform that localizes in the neuronal nucleus^20^. Therefore, the expression of the 0N4R isoform is expected to give rise to a nearly unique cytoplasmic distribution of Tau unless if fused to a NLS sequence. Hence, through the expression of Tau0N4R or Tau0N4R-NLS in KOTau neurons, it is possible to compare the effect of cytoplasmic with respect to nuclear distribution of Tau protein.

**Figure 3:**
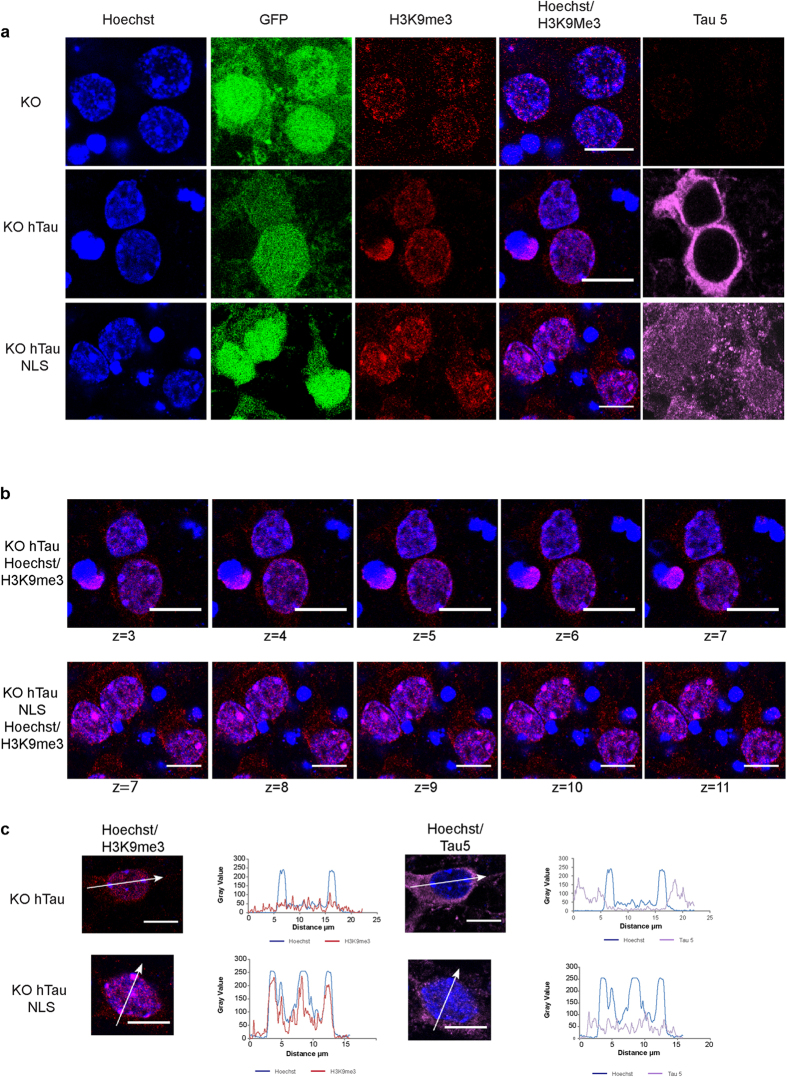
Nuclear hTau protein rescues H3K9me3 declustering in KOTau neurons. (**a**) Single confocal sections of nuclei from primary culture of KOTau neurons either non-infected (KO) or infected with lentiviral vectors encoding for hTau protein (KOhTau) or hTau protein fused to a nuclear localization signal (NLS) (KOhTauNLS) indicate that the clustered distribution of H3K9me3 in KOTau neurons is rescued in the presence of a nuclear localization of hTau protein. Tau5 antibodies were used to visualize total Tau protein and GFP fluorescence was used to visualize KOTau neurons. (**b**) Series of single confocal sections captured at 0.4 μm intervals displaying Hoechst/H3K9me3 merged images of KOhTau and KOhTauNLS nuclei indicate that all the chromocenters of KOhTauNLS, but not KOhTau, nuclei co-localize with clusters of H3K9me3. (**c**) Quantification of the fluorescence signals for Hoechst, H3K9me3 and Tau5 along the indicated line scans drawn across a confocal section of nuclei from KOhTau and KOhTauNLS neurons indicate that, whereas H3K9me3 is absent from chromocenters of KOhTau nuclei devoid of Tau5, H3K9me3 is present throughout the entire length dof chromocenters of KOhTauNLS nuclei displaying Tau5 labelling as previously observed in nuclei from WT neurons shown in Fig. 2d. Bars = 10 μm.

The presence of Tau protein was detected using pan-Tau (Tau5) antibody. As expected, no Tau protein was detected in KOTau neurons, which -in agreement with the results shown in Fig. 2- displayed a dispersed distribution of H3K9me3 (Fig. 3a). The expression of the non-NLS 0N4R isoform of Tau, which as expected displayed an almost exclusive cytoplasmic distribution, failed to restore the clustered distribution of H3K9me3 at chromocenters (Fig. 3a) and throughout all the confocal sections of a nucleus (Fig. 3b). In contrast, the expression of 0N4RTau-NLS, which displayed a cytoplasmic as well as a nuclear distribution, was able to almost fully restore the clustered distribution of H3K9me3 (Fig. 3a) with a majority of H3K9me3 co-localizing with chromocenters throughout all confocal sections (Fig. 3b). Line scans were drawn across chromocenters to quantify the relative intensities of the fluorescence signals for Hoechst (blue), H3K9me3 (red) and Tau5 (pink) (Fig. 3c). In KOhTau neurons that displayed only cytoplasmic Tau5 fluorescence, H3K9me3 fluorescence was scattered alongside the line scan with only casual low-intensity peak at chromocenters whereas in KOhTauNLS neurons, which displayed cytoplasmic as well as nuclear Tau5 labelling, the Hoechst and H3K9me3 intensity profiles overall overlapped, with H3K9me3 showing a maximum across all chromocenters (Fig. 3c). This analysis demonstrated the capacity of nuclear rather than cytoplasmic Tau to rescue the clustered distribution of H3K9me3 (Fig. 3c). Even though cytoplasmic localization of H3K9me3 detected in KOTau neurons remained visible in KOhTauNLS neurons, it was negligible compared with the nuclear H3K9me3 labelling in these neurons (Fig. 3c).

Overall, these results clearly indicate that in the nucleus, neuronal Tau protein plays a major role in regulating the clustered distribution of H3K9me3 associated with chromocenters.

### In AD brains, pathological phosphorylation of Tau protein is associated with an abnormal distribution of H3K9me3

The organization of neuronal PCH was then analysed in neurons from either Braak 6 AD or control brains by immunofluorescence and confocal microscopy using Hoechst staining of DNA, anti-H3K9me3 antibodies and the phospho-dependent AT8 antibody that recognizes Tau phosphorylated at Ser202/Thr205, an AD-relevant Tau epitope present from the early to late stages of Tau pathology.

**Figure 4:**
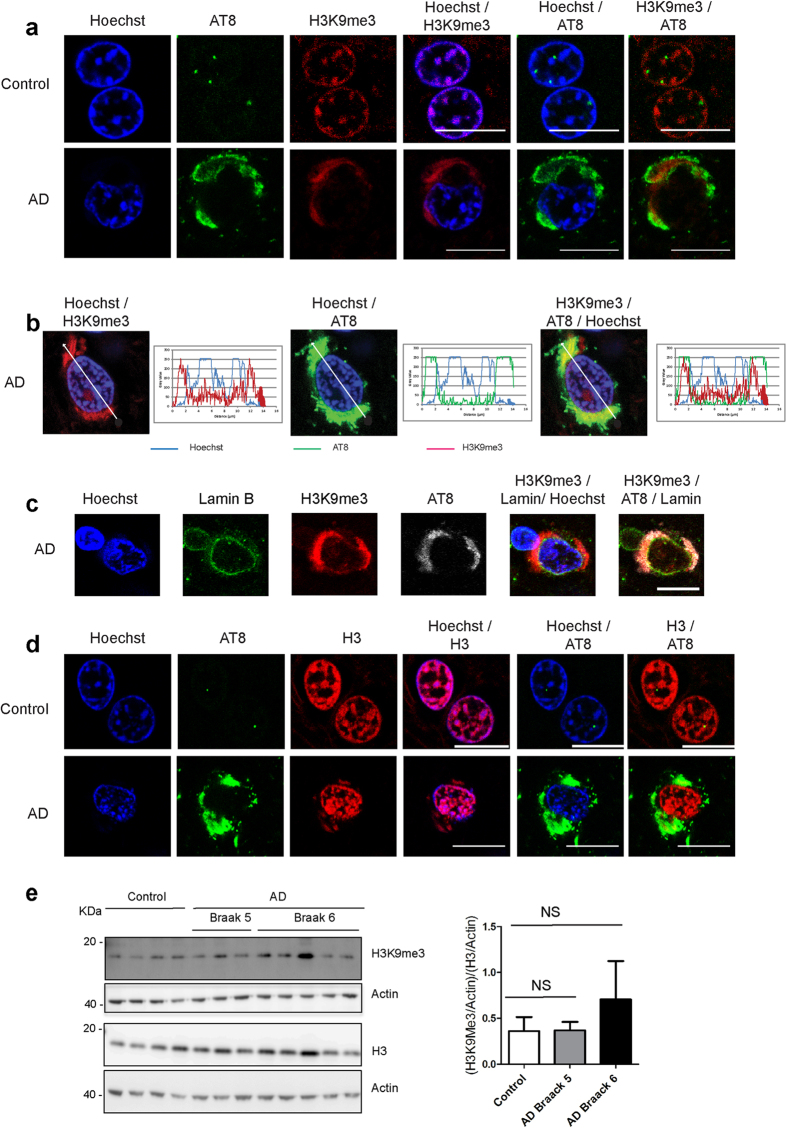
The clustered distribution of H3K9me3 is altered in nuclei from AD brains. (**a**) Single confocal sections of nuclei from control and AD brains indicate that the clustered distribution of H3K9me3 visible in nuclei of neurons from control brains is disrupted in nuclei of neurons from AD brains displaying AD-relevant phospho-dependent anti-Tau AT8 labelling. (**b**) Quantification of the fluorescence signals for Hoechst, H3K9me3 and Tau AT8 along the indicated line scans drawn across a confocal section of a nucleus of a neuron from an AD brain indicates that in neurons from AD brain H3K9me3 labelling is predominantly present outside the nucleus, co-localizing with AT8. (**c**) Single confocal sections of nuclei from AD brains indicating cytoplasmic localization of H3K9me3 and AT8 with respect to undisrupted lamin B labelling of the inner nuclear periphery. (**d**) Single confocal sections of nuclei from control and AD brains indicate that, in contrast to H3K9me3, the localization of H3 remains predominantly nuclear in neurons from control and AD brains displaying AD-relevant phospho-dependent anti-Tau AT8 labelling. (**e**) Western blot analysis of lysates from control (n = 4), AD Braak 5 (n = 3) and AD Braak 6 (n = 5) brain samples using antibodies against H3K9me3, H3 and actin indicate that the levels of H3K9me3 and H3 remain statistically unchanged in control and AD brains. The levels of H3K9me3 and H3 in each sample were estimated densitometrically with respect to the level of actin protein in the same sample. Data are means ± s.d. (**e)** One-way ANOVA test with NS (non-significant). Bars = 10 μm.

In nuclei of neurons from control brains, a clustered distribution of H3K9me3 was clearly observed to co-localize with densely Hoechst-labelled DNA structures (Fig. 4a), similarly to the co-localization of H3K9me3 with chromocenters observed in WT murine neurons (Fig. 2). As expected for neurons from control non-dement brains, AT8 labelling was almost completely absent with the exception of a few bright nuclear spots of AT8 detected next to, but not co-localizing with, H3K9me3 clusters (Fig. 4a). In neurons from AD brains that displayed as expected a strong AT8 labelling, the clustered distribution of H3K9me3 was strongly altered (Fig. 4a). Strikingly, in AT8 positive neurons from AD brains H3K9me3 labelling was undetectable in densely labelled DNA structures but detected in the cytoplasm within regions of intense AT8 labelling (Fig. 4a).

Figure 4b shows the relative fluorescence intensities of Hoechst (blue), H3K9me3 (red) and AT8 (green) in neurons from AD brains that confirm the exclusion of H3K9me3 from densely labelled DNA structures as well as its cytoplasmic localization within regions of intense AT8 labelling (Fig. 4b). Immunofluorescence labelling of lamin B, confirmed that H3K9me3 staining colocalizing with AT8 was localized outside the nucleus, which displayed a preserved lamin B distribution throughout the inner nuclear periphery (Fig. 4c).

The epigenetic H3K9me3 mark behaved as a subpopulation of total histone H3 specifically affected in neurons from AD brains. Indeed, the labelling of the total population of histone H3 (with an antibody that recognizes H3 independently of its post-translational modifications) remained mostly unchanged in neurons from AD with respect to control brains with a predominant nuclear distribution in association with DNA (Fig. 4d). Only a small fraction of total H3 (that could correspond to H3K9me3) displayed a cytoplasmic distribution within regions of intense AT8 labelling in AD neurons.

Western blot analysis of the total amount of histone H3 and H3K9me3 present in extracts from control and AD brains indicated that the total amounts of H3 or H3K9me3 or the corresponding H3K9me3/H3 ratio remained essentially unaffected in AD (Braak 5 and 6) with respect to the control brains (Fig. 4e), with only a slight increase in the H3K9me3/H3 ratio at a later stage of AD as observed in KOTau neurons (Fig. 2g). Therefore, the dispersion of H3K9me3 observed in neurons from AD brains cannot be considered a consequence of a decrease in the global amount of H3K9me3 or of the H3K9me3/H3 ratio.

Overall, the results indicate that AT8 positive neurons from AD brains displayed a phenotype similar to that induced by the lack of Tau protein observed in mice KOTau neurons with H3K9me3 totally absent from densely labelled DNA structures. Strikingly, the leakage of H3K9me3 outside the nucleus observed in KOTau neurons appeared to have been strongly exacerbated in neurons from AD brains in which almost all of H3K9me3 displayed a cytoplasmic distribution co-localizing with AT8.

### Tau protein regulates the recruitment of heterochromatin protein HP1α to chromocenters

**Figure 5:**
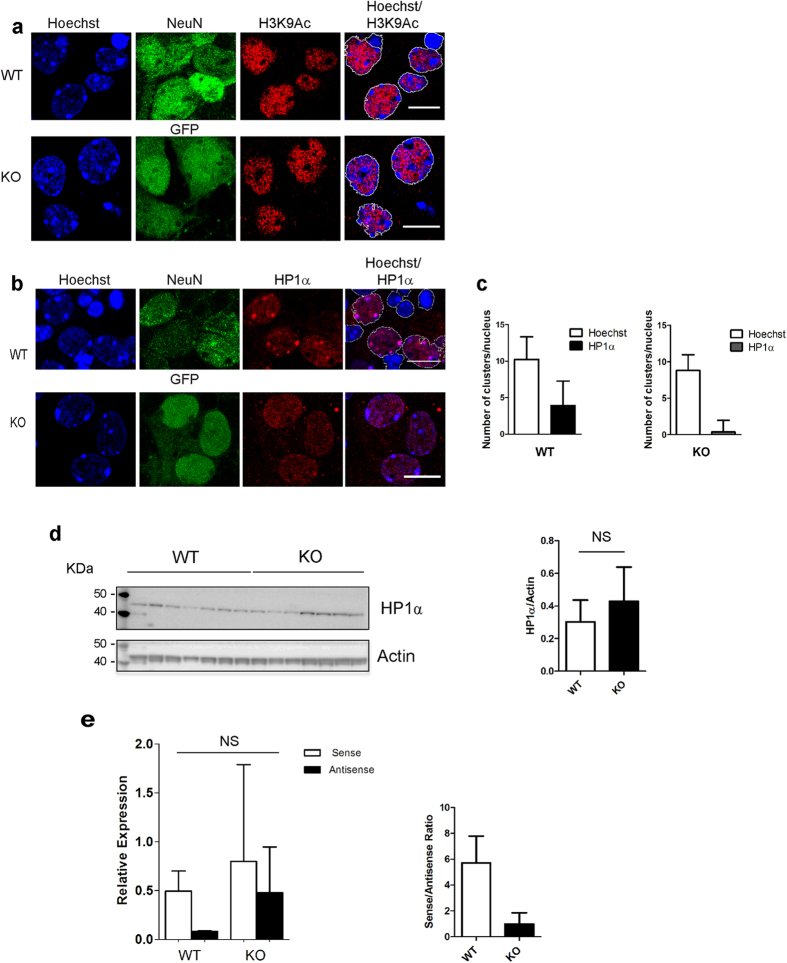
The clustered distribution of HP1α and the transcription of PCH are altered in Tau-deficient primary cultured neurons. (**a**) Single confocal sections of nuclei from primary culture of WT and KOTau (KO) neurons labelled with Hoechst, anti-NeuN or GFP and anti-H3K9Ac antibodies indicate that the loss of Tau protein does not affect the subnuclear distribution of H3K9Ac that remains excluded from chromocenters in WT and KOTau neurons. (**b**) Single confocal sections of nuclei from primary culture of WT or KOTau (KO) neurons (with nuclei outlined as relative to Hoechst fluorescence) labelled with anti-HP1α antibodies indicate that the clustered distribution of HP1α that partially co-localizes with chromocenters in WT neurons is disrupted in nuclei of KOTau neurons. (**c**) Quantification of chromocenters and HP1α clusters *per* nucleus after 3D reconstruction of nuclei from three independent WT and KOTau primary cultures of neurons (with 221 WT and 238 KOTau clusters analysed) indicate that whereas the number of chromocenters *per* nucleus remains unchanged in WT and KOTau neurons, the number of clusters of HP1α *per* nucleus is diminished in KOTau neurons compared with that in WT neurons. (**d**) Western blot analysis of lysates from n = 7 WT and n = 7 KOTau independent primary cultures using antibodies against HP1α and actin indicate that the total amount of HP1α remains statistically unchanged in WT and KOTau neurons. The level of HP1α in each sample was estimated densitometrically with respect to the level of actin protein in the same sample. (**e**) The relative expression levels of sense and antisense murine PCH non-coding RNAs determined by RT-qPCR after purification of RNAs from n = 2 WT and n = 2 KOTau independent primary cultures of neurons (with 10–20 embryos *per* WT culture) show a tendency towards the activation of the expression of the antisense non-coding RNA transcribed from murine major PCH satellite DNA in KOTau neurons, altering the ratio of sense to antisense PCH RNAs. Data are means ± s.d. (**c**,**d** and KOTau in **e**) ± s.e.m. (for WT in **e**). (**d**) Student’s t-test, (**e)** Two-way ANOVA with Bonferroni post-test, NS (non-significant). Bars = 10 μm.

Because the K9 residue of H3 cannot be simultaneously methylated and acetylated, these two epigenetic marks are mutually exclusive and display inverse distributions^21^. To determine whether the absence of H3K9me3 in chromocenters in KOTau neurons could be the consequence of an abnormal acetylation of H3K9 at these DNA regions, we carried out immunofluorescence and confocal microscopy analyses of the H3K9Ac distribution with respect to chromocenters in WT and KOTau neurons. As expected, H3K9Ac was observed to be excluded from H3K9me3-rich chromocenters in WT neurons but also from H3K9me3-deficient chromocenters in KOTau neurons (Fig. 5a). Therefore, the absence of H3K9me3 from KOTau chromocenters was not the consequence of an abnormal acetylation rate of H3K9 in these particular nuclear sub-structures.

Recruitment of heterochromatin protein 1α (HP1α) to pericentromeric DNA regions is necessary for the establishment of nucleation sites from which pericentromeric H3K9me3 initiates and propagates^16^ but is not necessary for maintaining PCH H3K9me3^22^. Immunofluorescence and confocal microscopy analyses of the clustered distribution of HP1α showed that in contrast to H3K9me3, a strong variation in the number of HP1α clusters/nuclei was observed among independent cultures from different animals, WT as well as KOTau, as determined using the chi-square test of homogeneity. However, notwithstanding strong variations observed among independent cultures, a partial clustered distribution of HP1α co-localizing with chromocenters was observed in WT neurons whereas HP1α displayed a completely dispersed distribution in KOTau neurons (Fig. 5b) with an average of 4.2 HP1α clusters/nuclei in WT neurons compared with 0.35 HP1α clusters/nuclei in KOTau neurons (Fig. 5c); whereas, the number of chromocenters/nuclei (corresponding to Hoechst clusters/nuclei) remained unchanged among WT and KOTau neurons as shown in Fig. 2e.

Even though the total amount of HP1α determined by western blot also varied from one culture to another, the average amount of protein HP1α observed in KOTau neurons remained similar to the amount observed in WT neurons (Fig. 5d), indicating that the absence of HP1α in PCH structures was not due to the absence of HP1α *per se*.

Overall, KOTau neurons appeared characterized by a failure to recruit HP1α to PCH clusters. However, intrinsic variations among independent cultures from different animals rendered differences between WT and KOTau neurons not statistically significant.

### Tau protein regulates PCH DNA transcription into non-coding RNAs

The transcription of PCH DNA sequences into non-coding RNAs, in both orientations, by RNA pol II is a feature conserved among most eukaryotic organisms from yeast to mammals^23^. Sense and antisense PCH RNAs have been shown to regulate chromocenter formation at the early stages of murine development^24^ and the sense PCH RNA under its single-stranded conformation was shown to be required for the initial targeting of HP1α to PCH in NIH-3T3 somatic mouse cells^25^.

The capacity of Tau protein to bind major satellite DNA sequences^7^ (Fig. 1b) and the recurrent presence of Tau either within or at the periphery of PCH (Fig. 1a) prompted us to investigate the potential role of Tau in regulating the expression of neuronal sense and antisense non-coding PCH RNAs. To this end, the presence of PCH RNAs was analysed by RT-qPCR in WT and KOTau neurons (Fig. 5e) using primers specific to either the sense or the antisense transcripts as described in^25^. Control qPCR assays in the absence of RT were carried out in parallel to verify that amplification was not the result of the presence of contaminating genomic DNA (data not shown).

In the case of WT neurons, both sense and antisense transcripts were detected with the sense RNAs being more abundant than antisense RNAs (Fig. 5e). In KOTau neurons, notwithstanding intrinsic variations between independent cultures from different animals, the relative expression of the antisense RNAs was observed as enhanced so that the average sense to antisense PCH RNA ratio diminished from 6 to 1.7 (Fig. 5e), thus decreasing the possibility for the sense RNA to be present under the single-stranded conformation required for the initial targeting of HP1α to PCH^25^.

### KOTau neurons display an abnormal accumulation of PCH DNA breaks

In *Drosophila*, mutants showing deficiency in H3K9me2/3 at chromocenters display high rates of double-strand breaks (DSB) at heterochromatin regions^26^. Based on our previous results indicating a role of nuclear Tau protein in neuronal DNA protection^5^,^6^, we examined here the potential role of Tau protein in protecting neurons from DNA breaks at PCH.

**Figure 6:**
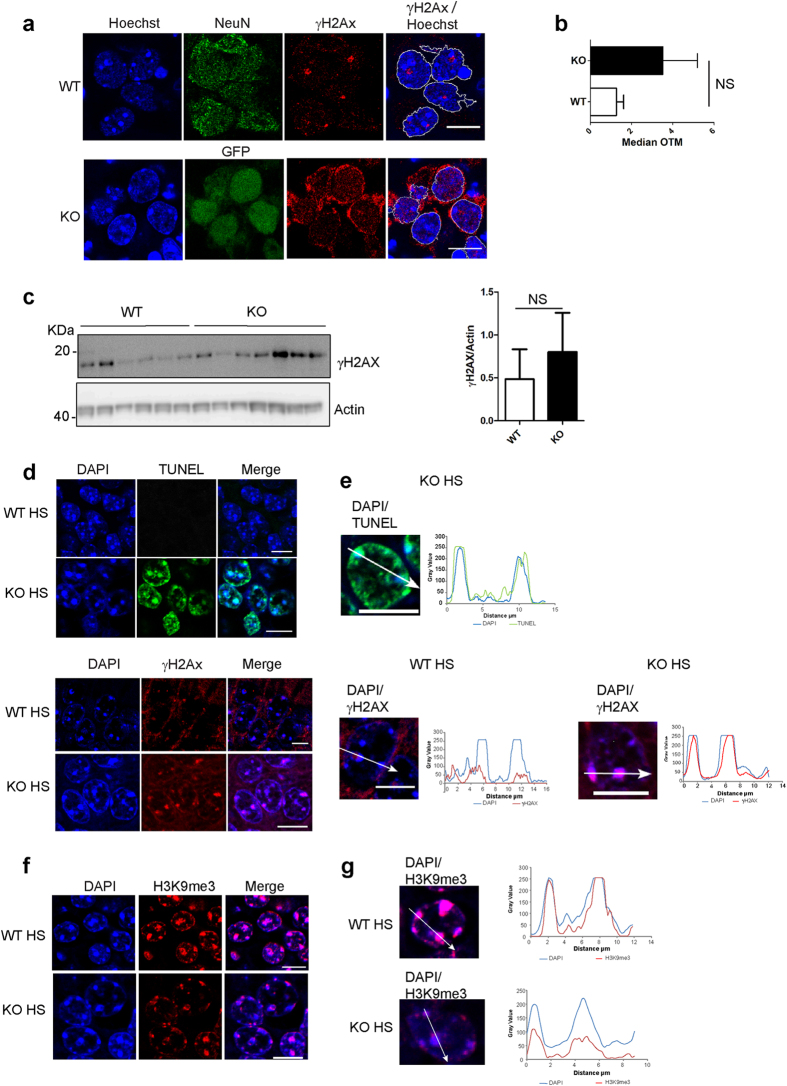
Tau deficiency is associated with an altered distribution of γH2AX and an accumulation of PCH DNA breaks in adult mouse hippocampus under stress conditions. (**a**) Single confocal sections of nuclei from primary culture of WT or KOTau (KO) neurons visualized with anti-NeuN antibodies or GFP fluorescence and labelled with Hoechst and anti-γH2AX antibodies indicate that the nuclear distribution of γH2AX at the periphery of chromocenters is affected in KOTau neurons. (**b**) The Comet assays show a tendency towards the accumulation of DNA damage in KOTau neurons compared with WT neurons. Each OTM (Olive tail moment) value is the median value of 150–200 cells from n = 7 independent WT and KOTau cultures. (**c**) Western blot analysis of lysates from n = 7 WT and n = 7 KOTau independent primary cultures. The level of γH2AX in each sample was estimated densitometrically with respect to the level of actin protein in the same sample. (**d**) Single confocal sagittal sections of hippocampus from WT or KOTau mice subjected to HS and labelled with DAPI and either TUNEL (green) or anti-γH2AX antibodies (red) show the accumulation of PCH DNA breaks within chromocenters of neurons from KOTau mice after HS treatment, which is correlated with the persistent co-localization of γH2AX within chromocenters of these neurons. (**e**) Quantification of the fluorescence signals for DAPI and TUNEL or DAPI and γH2AX along the indicated line scans drawn across a confocal section of nuclei from KOTau (KO) or WT neurons indicate that TUNEL and γH2AX labelling co-localize with chromocenters throughout the entire length of chromocenters of KOTau nuclei compared with chromocenters of WT nuclei, which remain mostly devoid of γH2AX. (**f**) Single confocal sections of hippocampus from adult WT or KOTau (KO) neurons indicate that under HS conditions the presence of H3K9me3 within chromocenters observed in WT adult neurons is diminished in nuclei of KOTau neurons as confirmed by (**g**) the quantification of the fluorescence signals for DAPI and H3K9me3 along the indicated lin scans drawn across chromocenters in a confocal section of nuclei from WT or KOTau (KO) neurons. Data are means ± s.e.m. (**b**) ± s.d. (**c**). (**b**) Mann-Whitney test. (**c**) Student’s t-test, NS (non-significant). Bars = 10 μm.

In *Drosophila* and mammalian cells, repair of PCH DSBs occurs through a two-step response^27^. First, the early DNA damage detection step that leads to the recruitment of DNA damage response proteins, such as the phosphorylated form of the histone variant H2AX (γH2AX) within the core of PCH structures, is followed by a second repair step that requires the relocation of the damaged sequences from the inside to the periphery of PCH, leading to the formation of long-lasting γH2AX foci positioned at the periphery of chromocenters^27^,^28^,^29^,^30^. Whereas foci of γH2AX positioned at the periphery of chromocenters could be detected in nuclei of WT neurons, they were absent from nuclei of KOTau neurons where dispersed γH2AX labelling was detected, concentrated in the cytoplasm and scattered as small foci in the nucleus (Fig. 6a). The monitoring of DNA damage by COMET assay (Fig. 6b) revealed a global increase in DNA damage in KOTau neurons compared with that observed in WT neurons, which did not reach statistical significance because of intrinsic variations between independent cultures from one KOTau mouse to another. Similarly to DNA damage, a global increase in γH2AX in KOTau neurons compared with that observed in WT neurons was detected (Fig. 6c), which again did not reach statistical significance because of intrinsic variations between independent cultures from different animals. Therefore, the absence of γH2AX foci at the periphery of chromocenters in KOTau neurons could not be considered a consequence of a decrease in these neurons of either the degree of DNA damage or of the level of γH2AX but rather of an abnormal localization of γH2AX reminiscent of what we have observed here for H3K9me3 in KOTau neurons.

Recruitment of γH2AX at sites of PCH DNA breaks followed by its relocation to the periphery of chromocenters is necessary for PCH DNA breaks to be repaired^28^,^29^,^30^. We therefore next examined whether the absence of γH2AX foci at the periphery of chromocenters in KOTau neurons was associated with an enhancement of DNA breaks specifically within these nuclear substructures. TUNEL assay was used to evaluate the subcellular distribution of fragmented DNA sequences at the single-cell level. No DNA breaks at PCH structures were detected by this technique in WT or KOTau neurons of embryonic origin under control conditions (data not shown). Subsequently, a TUNEL assay was used to evaluate the presence of DNA breaks within PCH structures in KOTau neurons of adult origin that were previously shown to display a high degree of DNA breaks under heat-shock (HS) conditions^6^. In agreement with these results, a strong TUNEL labelling was specifically observed within PCH of adult neurons from KOTau mice but not WT mice (Fig. 6d). Compared with WT adult neurons that, under HS conditions, displayed a weak γH2AX labelling within as well as at the periphery of PCH (Fig. 6d,e), adult KOTau neurons displayed a strong γH2AX labelling concentrated within PCH structures (Fig. 6d,e). Therefore, even though KOTau neurons appeared able to detect PCH DSBs and recruit γH2AX at sites of PCH DNA damage, they were unable to proceed to the second repair step that requires the positioning of the damaged sequences at the periphery of chromocenters, thus leading to an abnormal accumulation of DNA breaks within PCH.

The results shown in Fig. 6f,g indicated that under HS conditions, H3K9me3 staining in adult KOTau neurons was clearly different from the H3K9me3 staining observed in neurons from WT mice. In adult WT neurons, H3K9me3 completely overlapped the DAPI staining of chromocenters reaching the same intensity throughout the line scan. In contrast, in adult KOTau neurons, even though H3K9me3 staining was detected within the chromocenters, the intensity of H3K9me3 staining remained very low, never reaching the intensity of DAPI staining, similarly to the profile of H3K9me3 staining observed in KOTau neurons of embryonic origin (Fig. 2d). A diminished presence of H3K9me3 in PCH structures is in agreement with results obtained in *Drosophila* showing that the presence of H3K9me3 at PCH regions is necessary for PCH DNA repair to take place.

## Discussion

Using quantitative single-cell imaging and biochemical analysis of epigenetic marks and proteins we have analysed the organization of constitutive PCH in Tau-deficient neurons relative to that in Tau-expressing neurons. Here, we report that nuclear Tau protein regulates one of the main hallmarks of PCH structures: the clustered nuclear distribution of H3K9me3 and HP1α.

A loss of H3K9me2 and HP1α clusters has been recently described in *Drosophila* models of tauopathies corresponding to Tau transgenic flies expressing hTau either WT or as a pseudohyperphosphorylated form^11^. However, in this case, the loss of H3K9me2 and HP1α clusters was correlated with a global decrease in this epigenetic mark and protein and it was hypothesized to be the consequence of DNA damage induced by excessive oxidative stress^11^. Our results obtained using WT and KOTau mice primary cultured neurons, are indicative of a direct physiological role of endogenous Tau regulating neuronal PCH structures independently of the global level of H3K9me3 and HP1α. In contrast to *Drosophila* models of tauopathies^11^, overexpression of hTau in nuclei of embryonic murine KOTau neurons restored the clustered distribution of H3K9me3 that was lost not only in murine Tau-deficient neurons but also in human hippocampal neurons from AD brains. The loss of the clustered distribution of H3K9me3 observed in KOTau neurons of embryonic origin affected the localization of γH2AX with respect to PCH and rendered adult KOTau neurons unable to repair PCH HS-induced DNA breaks. These observations are in agreement with the role of heterochromatin and H3K9me3 in PCH DNA repair and γH2A localization described in *Drosophila* and *Neurospora crassa* respectively^21^,^31^.

The question regarding how endogenous Tau regulates H3K9me3 cluster formation at PCH structures is to be solved and remains a difficult issue because the mechanisms underlying PCH organization in mammals are for the most part unknown. However, the capacity of Tau protein to interact with PCH major satellite sequences and its distribution within or in the proximity of PCH structures described in this work suggests that Tau could play a role in addressing HP1α and therefore participate in the nucleation of H3K9me3 within PCH sequences.

In *S. pombe*, the initiation of PCH assembly and the establishment of H3K9me3 rely on the presence of non-coding RNAs transcribed from pericentromeric DNA as well as on RNAi components^32^. Even though the mechanism responsible of HP1 tethering and PCH assembly in mammals remains elusive, a role for the single-stranded conformation of non-coding PCH RNAs has been demonstrated^24^,^25^. In murine and human cells, these transcripts are of varying lengths and can exist in both orientations with the sense and antisense RNAs not necessarily present in equal amounts, which suggests different regulatory mechanism for each one of the two different transcripts^18^,^32^,^33^,^34^. Transcription of PCH RNAs has been shown to vary during development^24^,^35^, neuronal differentiation^36^, cell cycle^37^, in response to stress^38^,^39^ and in tumour cells^33^. However, little is known concerning the regulation of their transcriptional rate. The results presented in this work, suggest a role for Tau protein in regulating, as a repressor, the transcription of the PCH non-coding antisense RNA. By doing so, Tau would be a modulator of the ratio of sense to antisense PCH RNAs, affecting the equilibrium of the single- versus double-stranded conformations of these long non-coding RNAs in neuronal cells.

The atypical organization of PCH described in this work in KOTau neurons compared with that observed in WT neurons shares similarities with the organization of PCH observed in undifferentiated embryonic stem (ES) cells: a smaller number of H3K9me3 clusters/nuclei, enhanced diffuse distribution of HP1α and a higher degree of satellite repeat transcription^16^,^40^. The presence of such loose PCH structures in ES cells has been associated with the high degree of pluripotency characteristic of these cells. Genes present in pericentromeric-associated domains (PADs) are regulated differently in ES versus differentiated cells. Whereas PADs are transcriptionally active in ES cells, they become repressed as cells differentiate^41^. This behaviour is correlated, as the cell differentiates, with an increased association of PADs with chromocenters that progressively overlap with inactive repressed chromatin enriched in H3K9me3^40^. Therefore the establishment of a PCH exempt of H3K9me3, as observed in KOTau as well as in neurons from AD brains, might lead to the abnormal reactivation of the transcription of genes present within neuronal PADs and partly account for the previously reported increased expression in AD hippocampal neurons of heterochromatically silenced genes^11^. Interestingly, traits translating differentiation defects have been described in KOTau neurons^42^.

The inability to incorporate H3K9me3 within PCH observed in primary cultures of KOTau neurons was accompanied by the presence of scattered small clusters of H3K9me3 detected not only in the nucleus but also in the cytoplasm of these neurons, a situation that was also observed in the case of γH2AX but not in the case of H3K9Ac whose nuclear distribution remained unchanged in WT and KOTau neurons. This cytoplasmic distribution of H3K9me3 was exacerbated in AT8-positive neurons from AD brains in which H3K9me3 labelling was exclusively detected in the cytoplasm. Recently, aberrant cytoplasmic localization was described in hippocampal neurons from AD brains for another trimethylated form of H3, H3K4me3^43^, raising the issue concerning the potential effects of the presence of cytoplasmic histones in neuronal cells with respect to cellular signalling and innate immunity, as it has been reported in non-neuronal cells^44^.

To date, the hypothesis of a gain of pathological function, due to Tau hyperphosphorylation, oligomerisation and aggregation, in the establishment of AD has prevailed^45^. However, it cannot be excluded that the loss of Tau physiological function(s) might play a role in the development of AD. In this work, we have shed light on a new physiological function of Tau protein regulating the organization of neuronal PCH structures that are known to play important roles in regulating chromosome segregation, genomic stability and gene expression^12^,^13^,^15^,^26^,^46^. A similar loss of PCH organization was also observed in neurons from AD brains, strengthening the potential role of a loss of Tau function in the aetiology of AD.

## Materials and Methods

### Primary embryonic neuronal culture

Wild-type and knock-out Tau^19^ primary cortical neurons were prepared from 15 to 17 day-old mouse embryos as described previously^47^ and carried out in accordance with the approved guidelines. Briefly, cortex was carefully dissected out and mechanically dissociated in culture medium by triturating with a polished Pasteur pipette. Each embryo was dissected and platted individually.

### Human Brains

Human brain samples were obtained from the Lille Neurobank (presently the property of the French Research Ministry since 2008 under the reference DC-2000-642). The Lille Neurobank fulfills criteria from the French Law on biological resources, including informed consent, ethics review committee and data protection (article L1243-4 du Code de la Santé publique, August 2007).

### Antibodies

Primary antibodies used for immunoelectron microscopy, chromatin immunoprecipitation and immunofluorescence included, anti-trimethyl-HistoneH3Lys9 (07–442), anti-acetyl-HistoneH3Lys9 (06–942) and anti-γH2AX (05–636) from Upstate; anti-Tau1 (MAB3420), anti-NeuN (MAB377) and anti-phospho-Tau AT8 (MN1020) from Pierce; anti-H3 (ab24834) from Abcam; anti Tau5 (AHB0042) from Biosource; anti-HP1α (2HP-1H5-AS) from Euromedex; anti-lamin B (sc-6216) from Santa Cruz; mouse anti-NSs polyclonal antibodies^48^. Primary antibodies used for western blot included, anti-trimethyl-HistoneH3Lys9 (07–442) and anti-γH2AX (05–636) from Upstate; anti β-actin from Sigma, anti-H3 (ab24834) from Abcam; anti-HP1α (2HP-2G9-AS) from Euromedex. The secondary antibodies used for immunofluorescence and immuno-FISH were the Alexa 488-conjugated chicken anti-mouse (A21200), Alexa 555-conjugated donkey anti-mouse (A31570), and Alexa 555-conjugated donkey anti-rabbit (A31572) antibodies from Molecular Probes. The secondary antibodies used for western blot were the peroxydase horse anti-mouse (PI-2000) and peroxydase goat anti-rabbit (PI-1000) IgG antibodies from Vector laboratories.

### Immunoelectron microscopy

Cell cultures were fixed with 0.05% glutaraldehyde and 4% paraformaldehyde in 0.2 M Pipes buffer for 30 min at room temperature then at 4 °C for 1H. Cells were then incubated in phosphate-buffered saline containing 10% fetal calf serum, scraped, and centrifuged at 15,000 *g*. Pellets were soaked overnight in phosphate-buffered saline containing 2.3 M sucrose and 20% polyvinylpyrrolidone. Cells were rapidly frozen in liquid nitrogen. Frozen ultrathin sections were made with a cryo-ultramicrotome (Leica EM UCT) at a thickness of 85 nm with a cryochamber (Leica EM FCS). The sections were picked up on formvar-carbon-coated nickel grids. After a 30 min saturation in 2% bovine serum albumin, immunolabeling was carried out using Tau5, an anti-total Tau antibody, and an antibody recognizing H3K9me3. Tau5 labeling was revealed with a goat anti-mouse IgG gold conjugate (12 nm in diameter) (Jackson ImmunoResearch Laboratories) and H3K9me3 labeling was revealed with a goat anti-rabbit IgG gold conjugate (6 nm in diameter) (Jackson ImmunoResearch Laboratories). Negative staining of the ultrathin sections was carried out using 0.4% uranyl acetate and 1.8% methyl cellulose. Labeling was observed under a Zeiss 902 electron microscope.

### Chromatin immunoprecipitation

Chromatin immunoprecipitation experiments were carried out as previously described^49^. In Fig. 1b, semi-quantitative analysis of inputs and immunoprecipitated DNAs was performed by PCR as in ref. 50. Sense oligonucleotide 5′-TAT GGC GAG AAC CTG AAA-3′ and anti-sense oligonucleotide 5′-TTC ACG TCC TAA AGT GTG TAT-3′ were used as 5′ and 3′ primers respectively to amplify the murine major pericentromeric satellite DNA sequence and PCR conditions were as follows: 1 cycle of 94 °C for 5 minutes; 9 cycles of 94 °C for 30 seconds, 54 °C for 30 seconds and 72 °C for 30 seconds; 1 cycle of 72 °C for 10 minutes. In Fig. 1c, the immunoprecipitated DNA samples were quantified relative to input DNA using qPCR and calculated based on the formula: % of total input = [2^(∆CTinput−∆CTsample)^ × % of sample] using the same set of murine major satellite primers than for PCR.

### Immunofluorescence

Cells were fixed with 4% formaldehyde in phosphate-buffered saline (PBS) for 15 min and permeabilized with 1% Triton X-100 in PBS for 20 min. The cells were then incubated for 1 h at room temperature with corresponding antibodies diluted in PBS-5% bovine serum albumin. Cells were next washed with PBS and incubated for 45 min at room temperature with the corresponding secondary antibodies. Immunofluorescence in sagital brain sections was performed as described previously^51^.

### ImmunoFISH

ImmunoFISH was carried out as previously described^52^ except that fixed cells were kept overnight in 70% ethanol at 4 °C before permeabilization and treated for *in situ* DNA hybridization before immunofluorescence. Hybridization was carried out with a locked nucleic acid (LNA) fluorescent murine major satellite probe from Exiqon Cy3-LNA (5′-/TYE563/GCGAGGAAAACTGAAAAAGG-3′) as in ref. 25.

### Image analysis and quantification

Samples were analyzed at room temperature by confocal laser scanning microscopy using a AxioImager Z2 (Zeiss LSM710 confocal system). This system is equipped with a 63× lens, 1.4-numerical-aperture oil immersion lens (Plan Neofluor). For oil immersion microscopy, we used oil with refractive index of 1.518 (Zeiss). Images were captured in the direction of the z-axis corresponding to the optical axis of the microscope at 0.37 μm intervals with the z-axis going through the image planes. LSM Zen imaging software was used for image capture (512 × 512 pixels, 8 bit data). The images were analysed by the LSM5 Image browser or Image J software. For the quantification of the clusters we used the 3D object counter plugin from Image J^53^. In Fig. 1a, Tau-immunogold particles were identified applying the Analysis particle command of Image J software using the particle indicated by a bleu arrowhead as the lower size limit for the 12 nm gold particles recognizing the Tau5 antibody.

### Lentiviral vector (LV) infection

LVs vectors, encoding either for hTau0N3R or hTau0N3R-NLS, were amplified as previously described^54^. All viral batches were produced in appropriate areas in compliance with institutional protocols for genetically modified organisms according to the ‘Comité Scientifique du Haut Conseil des Biotechnologies’ (Identification Number 1285). For infection, round glass coverslips (18 mm diameter) were placed in 12 wells-plate and coated overnight at 4 °C with 0.1 mg/ml of poly-D-lysine and 0.02 mg/ml laminin (Sigma). Approximately 600,000 cells were plated on each glass coverslip. The cell culture were maintained at 37 °C in neurobasal medium (Gibco) supplemented with 2% B27 (Gibco), 200 μM L-glutamine and 1% antibiotic-antimycotic agent (Invitrogen). One week after plating, 200 ng of hTau0N4R LVs or hTau0N4R-NLS LVs were added per well. Seventy-two hours later, the cells were washed once with phosphate-buffered saline and fixed with 4% paraformaldehyde to perform immunocytochemistry.

### RT-qPCR of murine major satellite transcripts

Total RNA from primary cultured neurons was extracted with RNeasy plus kit (Qiagen). Genomic DNA was digested on the column by incubation with DNase 1 (Qiagen). The first-strand of sense and antisense murine major satelitte cDNAs were generated from 500 ng of total RNA using 1 μM of either sense (5′-AAATACACACTTTAGGACG-3′) or antisense (5′-TCAAGTGGATGTTTCTCATT-3′) specific primers respectively; the first-strand cDNA of reference gene Ppib was generated from 500 ng of total RNA using 1 μM of (5′-CTTCCGTACCACATCCATGC-3′) primer. The generated cDNAs were then amplified by qPCR using Absolute qPCR SYBR green ROX mix (Thermoscientific) as follows: 1 cycle (95 °C for 15 min); 40 cycles (95 °C for 15 sec.; 60 °C for 30 sec.; 72 °C for 30 sec.); 1 cycle (72 °C for 10 min). Relative expression was calculated with respect to the reference gene (Ppib) analyzed in the same samples.

### *In vivo* hyperthermia model

The *in vivo* mouse model of transient hyperthermic stress was performed as previously described^6^. Briefly, seven month-old homozygous female KO-Tau mice and littermate WT mice were anesthetized using xylazine (20 mg/kg) and ketamine (100 mg/kg) and maintained in a 37 °C environment for 30 min. The mice were then maintained at 37 °C (control (C) group) or heat stressed (HS group) by being placed in an incubator containing ambient air heated to 44 °C for 20 min. The rectal temperature of the mice was monitored every 10 min and did not exceed 41 °C.

### COMET assay

Comet assay was performed as previously described^5^.

### TUNEL staining

Terminal deoxyribonucleotidyltransferase-mediated deoxyuridine triphosphate nick end labeling (TUNEL) staining was conducted on tissue slices using the TUNEL Apoptosis Detection Kit (Millipore) according to the manufacturer’s instructions. Tissue slices were pretreated with low concentration of DNAse (1 μg/mL during 1 h) to perform positive controls. Brain slices from heat-stressed KO-Tau mice were incubated with DNAse-free RNAse (0.5 mg/mL, 3 h, Roche) prior to the TUNEL assay to eliminate TUNEL staining corresponding to HS-induced damaged RNA.

### Electrophoresis and immunoblotting

Lysates from WT and KOTau primary neuronal cultures were processed as previously described^47^.

### Statistical analyses

Statistical analyses were carried out using GraphPad Software. A two-tailed, unpaired Student’s t-test with α level = 0.05 was used to statistically analyse the data shown in Fig. 1c (Tau1 vs NSs), Figs 5d and 6b,c. Before the application of the Student’s t-test, the Shapiro-Wilk test of normality was used to verify that the data to be analysed were normally distributed and the F-test of equality of variances was used to verify that variances were not significantly different. In the case of Fig. 6b, which displays non-normally distributed data, a two-tailed non-parametric Mann-Whitney test was used with α level = 0.05 with non-significant difference observed.

The statistical analysis of the data shown in Fig. 2e was performed by applying the Chi-square test. The number of H3K9me3 clusters was compared with the number of chromocenters in nuclei from either WT or KOTau neurons using data from three independent WT or KOTau primary cultures from different animals after determining the homogeneity of the corresponding independent cultures. Alpha level = 0.05 and expected values in any category >5.

A one-way and two-way ANOVA test with α level = 0.05 was used to statistically analyse data from Figs 4e and 5e respectively.

### Ethics Statement

All animals were kept in standard animal cages under conventional laboratory conditions (12 h/12 h light/dark cycle, 22 °C), with ad libitum access to food and water. The animals were maintained in compliance with institutional protocols (Comité d'éthique en expérimentation animale du Nord Pas-de-Calais, no. 0508003). All animal experiment protocols were approved by the local Animal Ethical Committee (n°CEEA342012 on December 12, 2012 from CEEA Nord-Pas-de-Calais), standards for the care and use of laboratory animals, and the French and European Community rules.

## Additional Information

**How to cite this article**: Mansuroglu, Z. *et al*. Loss of Tau protein affects the structure, transcription and repair of neuronal pericentromeric heterochromatin. *Sci. Rep.* **6**, 33047; doi: 10.1038/srep33047 (2016).
